# Role of Telomeres and Telomeric Proteins in Human Malignancies and Their Therapeutic Potential

**DOI:** 10.3390/cancers12071901

**Published:** 2020-07-14

**Authors:** Stina George Fernandes, Rebecca Dsouza, Gouri Pandya, Anuradha Kirtonia, Vinay Tergaonkar, Sook Y. Lee, Manoj Garg, Ekta Khattar

**Affiliations:** 1 Sunandan Divatia School of Science, SVKM’s NMIMS (Deemed to be University), Vile Parle West, Mumbai 400056, India; stinaf2494@gmail.com (S.G.F.); dsouza.rebecca31@gmail.com (R.D.); 2 Amity Institute of Molecular Medicine and Stem Cell Research (AIMMSCR), Amity University Uttar Pradesh, Noida 201313, India; gpandya@amity.edu (G.P.); akirtonia@amity.edu (A.K.); 3 Laboratory of NF-κB Signaling, Institute of Molecular and Cell Biology (IMCB), 61 Biopolis Drive, Proteos, Singapore 138673, Singapore; vinayt@imcb.a-star.edu.sg (V.T.); sylee@imcb.a-star.edu.sg (S.Y.L.); 4 Department of Biochemistry, Yong Loo Lin School of Medicine, National University of Singapore (NUS), Singapore 117597, Singapore; 5 Department of Pathology, Yong Loo Lin School of Medicine, National University of Singapore (NUS), Singapore 117597, Singapore

**Keywords:** telomerase, telomeres, cancer, genomic stability, gene expression, therapeutic strategies

## Abstract

Telomeres are the ends of linear chromosomes comprised of repetitive nucleotide sequences in humans. Telomeres preserve chromosomal stability and genomic integrity. Telomere length shortens with every cell division in somatic cells, eventually resulting in replicative senescence once telomere length becomes critically short. Telomere shortening can be overcome by telomerase enzyme activity that is undetectable in somatic cells, while being active in germline cells, stem cells, and immune cells. Telomeres are bound by a shelterin complex that regulates telomere lengthening as well as protects them from being identified as DNA damage sites. Telomeres are transcribed by RNA polymerase II, and generate a long noncoding RNA called telomeric repeat-containing RNA (TERRA), which plays a key role in regulating subtelomeric gene expression. Replicative immortality and genome instability are hallmarks of cancer and to attain them cancer cells exploit telomere maintenance and telomere protection mechanisms. Thus, understanding the role of telomeres and their associated proteins in cancer initiation, progression and treatment is very important. The present review highlights the critical role of various telomeric components with recently established functions in cancer. Further, current strategies to target various telomeric components including human telomerase reverse transcriptase (hTERT) as a therapeutic approach in human malignancies are discussed.

## 1. Introduction

**Figure 1:**
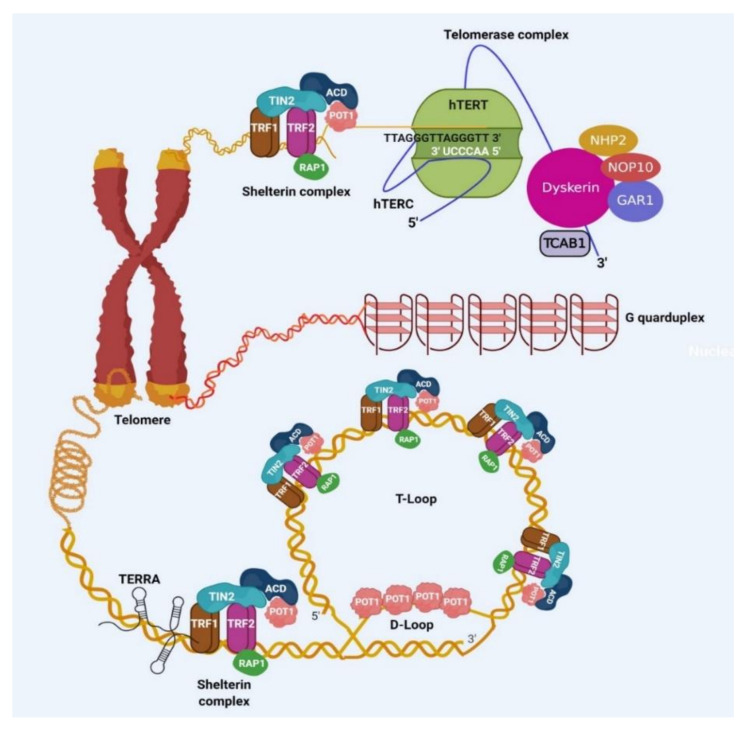
Schematic illustration of telomeric components. Shelterin complex comprising of the six members known as telomere repeat factor 1 (TRF1), telomere repeat factor 2 (TRF2), TRF interacting nuclear protein 2 (TIN2), repressor/activator protein 1 (RAP1), adrenocortical dysplasia protein homolog (ACD), and protection of telomere 1 (POT1), present at the telomeres. This complex directly binds to double-stranded telomeric DNA and is crucial for safeguarding the telomeres, as well as controlling th telomerase activity during elongation of telomeres. The telomerase is a complex represented by three major components: (a) telomerase reverse transcriptase (TERT), (b) RNA component (TERC), and (c) dyskerin complex containing NHP2, NOP10, TCAB1, and GAR proteins. This complex elongates telomeres. Telomeric DNA is transcribed to generate telomeric RNA called TERRA. Telomeric DNA can form secondary structures such as T-loop, D-loop and G-quadruplex.

Linear chromosome ends called telomeres are composed of 5′TTAGGGn tandem repeats ranging between 9–15 kb in length in humans [1]. Telomeres are essential in maintaining genomic stability and to execute this role, telomeres display various unique features which include telomeric structure, telomerase, shelterin complex, and telomeric repeat-containing RNA (TERRA) (Figure 1).

The double-stranded telomeric sequence is followed by single-stranded G rich overhang that is a few hundred bases long extending in 3′ orientation and is created post DNA replication [2]. Telomeric DNA has been implicated in the formation of various secondary structures like G-quadruplex (G4) DNA, T-loop, and D-loop [3,4,5]. G4 s are formed via Hoogsten hydrogen base pairing between four guanines. Telomeric G4 DNA can also form during replication when the duplex telomeric strand opens for the replication fork movement. Single-stranded G-rich overhang of telomeres has been observed to form G4s and at the telomeric overhang, they have also been proposed to prevent the action of nucleases on telomeres ensuring the stability of the genome [6,7]. T-loop and D-loop are formed when single-stranded overhang folds back and invades the double-strand telomeric strand and they protect telomeres from degradation [8]. The G4, T-loop, and D-loop are suggested to be mutually exclusive in occurrence (Figure 1).

Telomeres encounter the end replication problem which arises mostly due to the inability of DNA polymerase to replicate the ends during lagging strand synthesis [9]. Telomeres are elongated by enzyme telomerase which is a reverse transcriptase and minimally comprises of *TERC* (RNA component) which is a template to synthesize DNA and TERT (telomerase reverse transcriptase, protein) which is the catalytic subunit [10]. The maturation of active telomerase requires various accessory proteins like dyskerin, NHP2, NOP10, GAR1, TCAB1, reptin, and pontin [11]. Telomerase activity is present in stem cells, germ cells, and some immune cells while it is not detectable in human somatic cells mostly because of *TERT* transcriptional silencing during the development process while other components of telomerase including *TERC* are widely expressed. Reestablishing TERT expression exogenously is sufficient to restore telomerase activity in various primary human cells [12]. However, in some human cell types, *TERC* has also been shown to be a limiting factor for telomerase activity [13].

Due to the absence of telomerase activity in somatic cells, telomeres continue to shorten with every cell division [14]. The rate of telomere shortening varies across various cell types ranging between 15–200 bp for every population doubling [15,16,17,18]. This telomere shortening has been shown to correlate with the induction of senescence also referred to as replicative senescence (M1 stage) [14]. Critically short telomeres have been shown to activate DNA damage response (DDR) and it has been shown that the presence of approximately five DDR+ telomeres increased the probability of p53 driven senescence [19]. Inactivation of p53 and Rb tumor suppressors or expression viral oncogenes like SV40 T antigen has been shown to bypass senescence and cells continue dividing along with further telomere attrition and eventually reach crisis (M2 stage) which is characterized by severe genomic instability and apoptosis. Increasing telomere length experimentally can delay the crisis stage [20]. Furthermore, it has been observed that in human cells that have overcome the M1 stage by overexpressing the SV40-T antigen, the frequency of escape from the crisis is 10^−7^ [21]. Thus, the telomere-shortening pathway represents a very strong tumor suppressor mechanism along with p53 and Rb signaling. Approximately 85% to 90% of cancers (which include a wide range of different cancers) overcome this tumor suppression barrier by expressing hTERT and/or reactivating telomerase while the remaining use other mechanisms including the alternative lengthening of telomeres (ALT) [22,23].

The telomeric ends resemble DNA double-strand breaks and can potentially activate three DDR pathways which include classical nonhomologous end joining pathway (c-NHEJ), homology-directed repair (HDR) and alternate nonhomologous end joining pathway (A-NHEJ). Along with DDR, telomeres resembling double-strand breaks can signal activation of enzymes like ataxia–telangiectasia-mutated (ATM) kinase, ataxia–telangiectasia, and Rad3 related (ATR) kinase and poly(ADP-Ribose) polymerase (PARP1). DDR and kinases activation by telomeres is referred to as end protection problem and is essentially averted by the shelterin complex which comprises of six members that exhibit specific as well as overlapping functions in executing this role. Among them, telomere repeat factor 1/2 (TRF1/2) directly associate with double-stranded telomeric DNA and protection of telomeres 1 (POT1) interacts with single-stranded telomeric overhang. The remaining three proteins communicate with telomeres via protein–protein interactions. TRF-interacting nuclear protein 2 (TIN2) connects both TRF1/2 proteins and at the same time interacts with adrenocortical dysplasia protein homolog (ACD) (also known as TPP1), that simultaneously associates with POT1 [24]. Repressor activator protein 1 (RAP1) interacts with TRF2 along with DNA via structure recognition, but independent of sequence recognition [25,26] (Figure 1).

Telomeres encounter problems in replication due to structural hindrance arising from G4 formation in the DNA template during lagging strand synthesis [27]. TRF1 helps in evading this problem by recruiting BLM helicase to telomeres which has the ability to unwind G4 structures [28]. Regulator of telomere elongation helicase 1 (RTEL1) which is present in replication machinery also assists in removing G4 structures from the DNA template however it is recruited independent of TRF1 via its interaction with proliferating cell nuclear antigen (PCNA) [27,29]. TRF1 mutant which is unable to recruit BLM helicase or RTEL1 deletion results in the fragile telomere phenotype where gaps can be observed in the replicated telomeric DNA during metaphase. TRF2 plays an essential role in forming and protecting the T-loop at telomeres [30]. T-loop formed by TRF2 further inhibit recruitment of Ku70/80 and Mre11-Rad50-Nbs1 (MRN) complex thus repressing c-NHEJ pathway activation (which results in telomere overhang processing followed by telomere fusion) and ATM kinase activation (which results in accumulation of damage responsive proteins like 53BP1, gammaH2AX (γH2AX), Rad17, ATM, and Mre11 at telomeres called as telomere dysfunction induced foci or TIFs and cell cycle arrest), respectively [31]. Further TRF2 also recruits RTEL1 during S phase of the cell cycle to promote T-loop unwinding thus promoting telomere replication [32]. RAP1 enhances the selectivity of the TRF2 towards the telomeric DNA [33]. Further RAP1 along with POT1 have independently been shown to repress HDR at telomeres [34,35]. POT1 represses the ATR-mediated repair pathway by interfering with the recruitment of replication protein A (RPA) [31]. TIN2 stabilizes TRF1 by preventing poly(ADP-ribosyl)ation of TRF1 by tankyrase and also prevents its ubiquitin-dependent proteolysis by competing with SCF^FBX4^ [36,37]. Further TIN2 has a direct structural role in telomere protection. It connects ACD–POT1 dimer to other parts of the shelterin like a bridge [24]. Thus, it has a role in promoting ATR and ATM repression by stabilizing other shelterin components to telomeric DNA [38]. In addition, TIN2 and ACD play an important role in telomere maintenance by recruiting telomerase to telomeres [39,40]. Additionally, ACD–POT1 together increases the processivity of telomerase [41,42].

Shelterin components and telomerase also play a critical role in telomeric 3′ overhang formation [43]. Leading and lagging strand synthesis during DNA replication generate very different telomeric ends. At lagging end telomeres, 3′ overhang is formed due to the inability of DNA polymerase α/primase complex to initiate DNA synthesis at the very end of linear chromosomes while leading end telomeres are blunt [44]. At the leading end, 3′ overhang has to be generated. Mechanistically it has been demonstrated that Apollo 1 nuclease is recruited by TRF2 initiates 5′ end resection at leading end telomeres which is blocked by POT1 at lagging end telomeres. Subsequently, Exo1 nuclease is recruited which performs hyper-resection, transiently extending the overhang length. This is followed by fill-in synthesis where POT1 associated with CST complex (Ctc1, Stn1, Ten1) to recruit DNA polymerase α/primase complex thus generating appropriate length overhangs in both the duplexes [43].

Adjacent to telomeric repeats in chromosomes are regions called subtelomeres which have been implicated in transcriptional regulation as well as telomeric chromatin organization, protection, and regulation. RNA polymerase II can initiate transcription in this region progressing towards telomeres and generates long noncoding RNA named TERRA [45,46]. Transcription factors like CCCTC-binding factor (CTCF) and nuclear respiratory factor 1 (NRF1) have been proposed to regulate telomeric transcription [47,48]. TERRA has been proposed to function in cis (on telomeres from where they have been transcribed) as well as in trans (remotely working on telomeres while being transcribed from different telomeres) [47,49,50]. TERRA has been shown to function in end protection, telomeric replication, and telomerase recruitment [47,51] (Figure 1).

Telomere dysfunction can arise due to telomere uncapping (defined as critically short telomeres or removal of telomere protective factors like shelterin components) or crisis and various studies have linked this phenomenon to cancer [47,52,53]. Cancer genomes show marked genomic instability very similar to that observed due to the telomere crisis [54]. Telomere length also plays a critical role in cancer as both long and short telomeres may help in promoting cancer at different stages of development. In this review, we highlight the role of various telomeric components in cancer followed by how these components are targeted for their potential therapeutic application in cancer.

## 2. Importance of Shelterin Complex in Cancer

Shelterin complex plays an essential role in telomere protection and altering its function is associated with telomere deprotection which is an important source of genomic instability. Since one of the hallmarks of cancer is genomic instability, investigating the role of shelterin components in cancer becomes very important. Altered expression and several mutations in shelterin components have been reported in various cancers (Table 1).

Several groups have shown that TRF1, TRF2, RAP1, POT1, and TIN2 are highly overexpressed in hepatocellular carcinoma, lung adenocarcinoma, gastric carcinoma, leukemia, renal cell carcinoma, prostate cancer and suggested their role in promoting cancer [55,56,57,58,59,60,61,62,63,64,65]. Increased expression of TRF2 has been proposed in promoting tumorigenesis mechanisms like initiation, progression, migration, metastasis, angiogenesis, and immunosurveillance [66,67,68,69]. Similarly, increased expression of TRF1 has been reported to promote cancer stemness and telomere maintenance [70,71]. While in some cancers reduced expression of shelterin proteins have been reported [72,73,74,75,76]. Differential expression patterns of telomeric proteins have been implicated in the pathogenesis of B cell-chronic lymphocytic leukemia (CLL) where the expression of *TRF1* and *POT1* were reduced more than two-fold whereas *ACD* and *RAP1* showed increased expression [77,78].

Germline and somatic mutations in some of the shelterin component genes have also been associated with various cancers [79]. Among all, *POT1* has been reported to be one of the most commonly mutated genes with recurrent mutations observed in familial melanoma, glioma, CLL, mantle cell lymphoma, cardiac angiosarcoma in Li Fraumeni like syndrome, colorectal cancer and Hodgkin lymphoma [79,80,81,82,83,84,85,86,87]. POT1 protein has two major domains: telomeric ssDNA binding domain and ACD interacting domain. Different mutations observed in POT1 have been segregated into three groups based on their structure and functional studies: missense mutations in the ssDNA binding domain; missense mutations in ACD interacting domain and; other disruptive variants which can be nonsense, splice acceptor or donor and frameshift mutations. The most consistent telomere phenotype is progressive telomere lengthening which has been proposed to either bypass replicative senescence checkpoint thus promoting cancer development or result in replication defects in telomeres which in turn increases telomere fragility subsequently destabilizing genome which can accumulate further mutations to result in cancer [88]. Somatic mutations in *POT1* have also been found in CLL as well as in cutaneous T-cell lymphomas (CTCL) where telomere abnormalities and genomic instability were also observed further supporting its role in malignancy [89,90]. Germline mutations in *ACD* and *RAP1* have also been reported in various familial cancers [84,91].

Genetically modified mouse models for shelterin components have also been employed to understand their role in cancer. Ectopic expression of TRF2 in the skin has been shown to accelerate tumorigenesis and genetic deletion of *TRF1* has been shown to impair the growth of *p53*-null *K-Ras^(G12 V)^*-induced lung carcinomas thus increasing mouse survival independently of telomere length [71,92,93]. Loss of *Rap1* has also been reported to accelerate Myc and MMTV driven lymphomagenesis and breast tumorigenesis, respectively [94]. Additionally, TRF2 and Rap1 have also been reported to have extra telomeric effects which have been suggested to have putative roles in tumorigenesis. TRF2 has been shown to regulate angiogenesis by directly regulating the expression of platelet-derived growth factor receptor-β [67]. TRF2 has also been found to transcriptionally repress cyclin-dependent kinase CDKNIA (p21/CIP1/WAF1) expression through recruiting REST-coREST-LSD1-repressor complex. This repression was further reported to overcome G2/M arrest caused due to drug-induced DNA damage activation in cancer cells [95]. Rap1 has also been reported to associate with subtelomeric gene promoters as well as promoters of genes involved in cellular metabolism, cell-to-cell adhesion and cancer [96,97,98]. Additionally, Rap1 has been shown to regulate inflammation via interacting with IKK complex and thus affecting NF-ĸB activity which also has an important role in cancer [99].

## 3. Importance of Telomerase in Cancer

Telomerase is detected at a frequency of 80–90% in cancer tissues making it one of the most common tumor-associated markers [22,97,100]. Telomerase plays a very crucial role in cancer cells by maintaining telomere length which is essential for the continuous proliferation of cancer cells [101]. Telomerase silencing acts as a primary barrier against cancer which is achieved by *TERT* transcriptional silencing or is alternatively spliced to non-telomerase coding variants [102,103,104]. Several cancers harbor various genomic alterations in *TERT* with most of them correlating with *TERT* expression level, telomerase activity, and telomere length [105]. In addition to the role of TERT in reestablishing telomerase activity that elongates telomeres, recent observations also reveal multiple oncogenic activities associated with telomerase subunit TERT in cancer which may or may not require catalytically active telomerase [106]. These include signaling pathways that influence cancer development and progression. TERT has been found to regulate vascular endothelial growth factor (VEGF) expression, NF-κB and Wnt/β-catenin signaling cascade, repress TNF-α-mediated gene expression and ROS-mediated intracellular pathways, processes intricately involved in carcinogenesis [107,108,109,110]. TERT has been shown to promote MYC dependent lymphomagenesis independent of its catalytic activity [111]. This study provided the first genetic evidence for the noncanonical role of TERT in oncogenesis. Furthermore, telomerase interacts with RNA polymerase I and III subunits and stimulates rRNA and tRNA transcription and plays an important role in oncogenesis [112,113]. TERT has also been observed to interact with the SP1 transcription factor and contribute to angiogenesis [114]. Thus, telomerase/TERT contributes to several hallmarks of cancer which may be dependent or independent of its catalytic activity [115]. Genetic alterations regulating human *TERT* gene in cancer and cohort studies are described below:

### 3.1. TERT Promoter Mutations (TPMs)

Multiple transcription factors including Myc, NF-kB, β-catenin have been identified to regulate *TERT* transcription however how constitutive activation of *TERT* transcription in multiple cancer cell types is achieved remains an intriguing area of investigation. In 2013, two research groups discovered the presence of noncoding TPMs in human melanomas [116,117]. Barthel and colleagues analyzed the TCGA dataset from the pooled cohort covering 31 different cancer types and reported 27% of all analyzed samples showed TPMs [105]. Depending on the tissue type, cancers can be categorized as harboring low (<15%), intermediate (15–50%), or high (>50%) frequency of TPMs (% of the number of tumors showing TPMs within the total number of samples analyzed). For example Killela and colleagues reported TPMs frequency for gliomas (including 11 subtypes): 51%, hepatocellular carcinoma: 44.2%, urothelial carcinoma of bladder: 66.6% [118]. Vinagre and colleagues reported TPMs frequency for central nervous system cancers: 43%, bladder cancer: 59%, and skin cancer (melanoma): 29%, follicular cell-derived thyroid cancer: 10% [119]. Cancers from intestinal tissues or blood cells were found to have a very low frequency of undetectable TPMs [118]. Thus, TPMs have been observed to be rare in tumors originating from highly regenerative tissues while being more frequent in cancers originating from low rates of self-renewal like brain, bladder, and liver. This further suggests that TPMs may be advantageous for cancers initiating from tissues with low or undetectable *TERT* expression while providing no selective advantage to cancers arising from stem cell compartment. The presence of TPMs was elegantly investigated in melanoma progression beginning from benign lesion > intermediate lesion > melanoma in situ > invasive melanoma to metastatic melanoma by Shain and colleagues [120]. They observed the presence of TPMs in intermediate lesions and melanomas in situ occurring in 77% of these neoplasms thus suggesting them to be early events in tumorigenesis process. TPMs have been observed as early genetic alterations in bladder cancer, hepatocellular carcinoma, melanoma, basal cell carcinoma, squamous cell carcinoma, and oligodendroma [121,122,123,124,125,126].

TPMs are mutually exclusive and heterozygous showing monoallelic expression from the allele having TPM [116,127]. Majority of these mutations are present at two positions −124 bp (C>T) and −146 bp (C > T) relative to the transcription start site of *TERT* gene also known as C228T and C250T (according to genomic location chr5, 1,295,228 C > T and 1,295,250 C > T) respectively [116,117]. The prevalence of C228T is higher than C250T in almost all cancer types except melanoma and nonmelanoma skin carcinomas where they have been reported to be equally frequent when compared with total *TERT* promoter mutated samples [128,129]. Mechanistically, C228T or C250T mutations generate a de novo binding site for ETS transcription factors, GABPA and GABPB1 (GA-binding proteins), activating *TERT* transcription, and telomerase activity [130]. Also, C228T and C250T have been reported to be functionally distinct and show context-dependent regulation. For example, noncanonical NF-κB subunit, p52, has been observed to dimerize with ETS1/2 factors selectively on the C250T-mutant *TERT* promoter thus activating *TERT* transcription [131]. Apart from C228T and C250T mutations, a few other mutations also have been found to regulate TERT. A mutation at the MYC binding site at the TERT promoter has been reported in approximately 8% of patients with renal cell carcinoma [132]. This complex is known to function as a repressor and mutation in its binding site on TERT promoter is suggested to derepress the TERT promoter.

TPMs are mostly associated with higher *TERT* expression when compared with tumors having no TPMs. Vinagre and colleagues reported that TPMs in thyroid cancer were significantly associated with increased *TERT* expression while tumors of the central nervous system showed no significant association when compared with wild type *TERT* promoter bearing tumors [119]. Fredriksson and colleagues reported that TPMs in bladder cancer, glioblastoma, low-grade glioma, and thyroid cancer was significantly associated with increased *TERT* expression while melanoma showed no significant association between mutation status and expression when compared with wild type *TERT* promoter bearing tumors [133]. Additionally, engineering TPM in human embryonic stem cells led to constitutive expression of *TERT* and telomerase activity bypassing developmental silencing even after terminal differentiation, and the mutation could immortalize the cells [13]. TPMs have also been found to be associated with increased telomerase activity in primary glioblastoma (GBM) patient samples as well as in urothelial and GBM cell lines (comparison between wild type vs mutant *TERT* promoter) [134,135]. On the contrary to the association of TPMs with *TERT* expression and telomerase activity, its association with telomere length has been inverse. Tumors with TPMs have shorter telomeres when compared with tumors having wild type *TERT* promoter [136,137]. Additionally, telomeres were also found to be shorter in tumors with TPMs when they were compared with normal matched control tissues in gliomas, clear-cell renal cell cancer, and melanoma [132,133,134,135,136,137,138,139].

TPMs have also been reported to be associated with specific clinical and phenotypic subtypes and disease outcome. TPMs associate with adverse disease outcomes in most of the malignancies. In melanoma, TPMs have been shown to associate with poor disease-free and melanoma-specific survival and if simultaneously present with *BRAF/NRAS* oncogenic mutations, it could predict worst disease-free progression and melanoma-specific survival [140]. Similarly, TPMs have been shown to be associated with the self-renewal ability of GBM cells and their clinical aggressiveness [141]. In papillary thyroid cancers, TPMs together with *BRAF* alterations cooperatively outlined most aggressive subtypes exhibiting the highest recurrence, distant metastasis, and mortality [142]. In bladder cancer, TPMs are also reported to associate with increased mortality and disease recurrence [143]. These studies highlight the prognostic significance of TPMs and their utility as a clinical biomarker.

### 3.2. TERT Gene Amplification

The amplification of oncogene is one of the most common events in various human malignancies [144]. It has been reported that *TERT* expression was dependent on gene-dosage and haplo-insufficient for telomere maintenance in human cells in vivo [145]. Thus, an increase in *TERT* copy number could result in increased expression of *TERT* thus reestablishing telomerase activity in cancers. *TERT* copy number amplification has been observed in various cancers including skin, thyroid, and breast cancer where it was found to be associated with increased *TERT* expression and was positively correlated with worse clinical outcomes [146,147,148,149]. However, there are reports where no correlation could be observed between amplification of *TERT* gene and *TERT* mRNA expression, telomerase activity or telomere length suggesting there may be some additional molecular events required for this association and thus require further investigation [150].

### 3.3. Rearrangement of TERT Locus

Chromosomal rearrangement of *TERT* locus was first reported in immortalized fibroblast cell lines where translocation of *TERT* to a different locus resulted in its transcriptional reactivation thus reestablishing telomerase activity and stabilizing telomere length [151]. Translocation of *TERT* locus in B-cell malignancies were also observed to correlate with higher *TERT* expression and increased telomerase activity and suggested to contribute to B-cell lymphomagenesis [152,153]. With the advent of genome sequencing, the rearrangement of *TERT* locus has also been identified to occur in various cancers types [105]. Particularly in neuroblastoma (cancer of immature nerve cells called neuroblasts commonly occurring in infants and young children), *TERT* locus rearrangements have been reported to be frequent and segregate with aggressive tumors [154,155,156]. The rearrangement specifically placed *TERT* locus close to super-enhancers and occurred exclusively to *MYCN* amplification (which is a transcriptional activator of *TERT* thus can establish active telomerase for telomere length maintenance) or α-thalassemia mental retardation X-linked protein (*ATRX*) deletions (which result in telomere length maintenance by ALT activation) suggesting different subgroups employ different genetic alterations to maintain telomere length and continue proliferation [157,158,159].

### 3.4. TERT Transcription through Telomere Position Effect-Over Long Distances (TPE-OLD)

TPE is a mechanism where genes proximal to the telomeres are transcriptionally silenced and it depends on the telomere length and distance from telomeres [160]. As telomeres progressively shorten with cell divisions, expression of subtelomeric silenced genes become active and this phenomenon has also been linked to senescence as well as aging. Similarly, there is another phenomenon called TPE-OLD which modulates the expression of genes that are located at a certain distance from telomeres. It was observed that young normal human cells that have long telomeres form telomere loops in the region located nearby to the *TERT* locus in a way that TPE-OLD genes come in direct proximity of the telomere [104,160]. This telomere loop was observed to hinder transcription of *TERT* and nearby genes, but in aged cells with shortened telomeres, the repressive loop is disrupted which opens the closed chromatin to induce transcription of *TERT* and TPE-OLD genes. However, through TPE-OLD in shorter telomere containing genome, only the first exon of *TERT* is transcribed as mRNA which lacks reverse transcriptase domain and thus is incapable to reestablish telomerase activity. Since short telomeres are a common genomic feature of cancer cells, TPE-OLD may contribute to *TERT* expression/telomerase activity. However, the role of TPE-OLD and its cooperation with other mechanisms in *TERT* activation during cancer requires further investigation.

### 3.5. Oncoviral DNA Insertions at TERT Locus

Activation of telomerase is one of the crucial mechanisms for oncogenic-viral infected tumorigenesis [161]. It has been observed that viral proteins derived from several viruses such as Hepatitis B virus (HBV), Epstein–Barr virus (EBV), human papillomavirus (HPV), Cytomegalovirus (CMV) act as cofactors to stimulate *TERT* transcription. It was demonstrated that the HBV enhancer-containing DNA fragment inserts into the 5′ regulatory region 1.6 kb upstream of the *TERT* transcription start site to drive *TERT* transcription in HCC cells [162]. Genome-wide sequencing analysis of HCC (HBV-positive) showed that the *TERT* locus has the highest HBV DNA insertion with maximum integration breakpoints in the *TERT* promoter region. At least one viral gene enhancer and/or promoter has been shown to be present in almost all the integrated DNA samples consequently increasing TERT expression [163,164]. In the case of HBV-negative HCC, adeno-associated virus type 2 may be engaged in the oncogenic related processes [165]. Other than HBV, there is little information on the genomic interaction between the host *TERT* and other oncoviruses. In a screening of various oncoviruses like HPV, BKV, and EBV in various types of cancers, Chen and colleagues have confirmed the insertion at *TERT* locus, but the integration of breakpoints was in regions other than *TERT* locus [163]. In squamous cell carcinoma of the oral cavity, it has been demonstrated that HPV DNA targeted the *TERT* locus for integration [166].

### 3.6. Alternative Splicing of TERT

*TERT* RNA undergoes alternative splicing regulation and more than 20 splice variants have been identified [167]. Only full-length mRNA of *TERT* contains all 16 exons including reverse transcriptase domain coding exons (exon4– exon11). All the alternatively spliced variants lack reverse transcriptase activity and thus cannot elongate telomeres [168,169]. Alternative splicing of *TERT* has also been proposed to be involved in telomerase silencing during the development process and as one of the putative mechanisms involved in telomerase activation in cancer cells [103,170]. Due to TPE-OLD, telomere shortening results in *TERT* transcription activation where only exon 1 containing mRNA is produced [104]. It is proposed that during cancer development some unknown additional event changes the alternative splicing pathway to produce full-length *TERT* and reestablish telomerase activity. For example, recently NOVA1 was reported to be an important regulator of *TERT* alternative splicing in non-small cell lung carcinoma (NSCLC) [171]. Inhibition of NOVA1 resulted in the production of noncatalytic alternatively spliced *TERT* variants that resulted in reduced telomerase activity in cells and progressive telomere attrition. Further investigation is required in this field to understand the molecular and cellular events involved in alternative splicing regulation of *TERT* in normal development in various tissues and during cancer initiation and progression.

### 3.7. Cohort Studies and Future Directions

Recently, a worldwide effort from the interdisciplinary group of scientists with 744 affiliations altogether analyzed whole genome sequencing dataset of 2500 matched tumor and control samples comprising 36 different tumor types deposited within the ICGC/TCGA Pan-Cancer Analysis of Whole Genomes (PCAWG) Consortium to understand various genomic aspects of cancer [172]. While they confirmed the importance of telomerase activation via the above described mechanisms as well as alternative lengthening of telomeres, several interesting findings have emerged from the analysis of telomere maintenance mechanisms in cancer. They classified tumors into four clusters based on 12 parameters which are: sequence counts of nine variants of the telomeric core hexameric sequence, the number of telomere-like sequences ectopically inserted within the genome, the number of breakpoints in genome and telomere length as a ratio between tumor and normal. The group observed that *TERT* genetic alterations activating telomerase and ALT (mostly *ATRX* or *DAAX* deletions) appeared as two separate clusters. The third cluster comprised tumors with *RB1* alterations or *ATRX* structural variants where both were mutually exclusive in occurrence. Forth and the largest cluster comprised mixture of either *TERT* or *ATRX/DAAX* or *RB1* genetic alterations occurring exclusively to each other, but clustering separately based on 12 parameters suggests the existence of additional unknown telomere homeostasis mechanisms which tumors must overcome to achieve their fate. It would be extremely interesting to find those mechanisms which make this cluster. Further, the study also proves altered telomere maintenance mechanisms in tumors originating from tissues with low replicative potential while being more intact in tumors originating from tissues with high replicative capacity [172,173].

## 4. Role of TERRA in Cancer

Most cancer cells rely on telomerase for maintaining telomere length, a subset of telomerase negative cancers employ the ALT pathway to maintain telomere length [174,175]. ALT has been observed to range from 25% to 60% in sarcomas and 5% to 15% in carcinomas [176]. Various recent findings report elevated levels of TERRA in ALT positive tumors where it has been observed to play an important role in telomere maintenance [177]. TERRA has been shown to inhibit telomerase activity in vitro [178]. TERRA molecules contain 5′-UUAGGG-3′ repeats close to their 3′-end and they are complementary to the template sequence of *TERC* and it has been observed that TERRA interacts with *TERC* by base pairing with these repeats. TERRA also interacts with TERT independent of *TERC,* but instead of acting as a substrate, it acts as a natural ligand and as a direct inhibitor of telomerase enzyme activity. Therapeutic exploration of TERRA-mediated telomerase regulation against cancer appears to hold huge potential. Additionally TERRA expression is downregulated in advanced stages of various cancers suggesting that lower TERRA expression may promote telomerase-mediated elongation of telomeres in cancer cells [179]. However, further investigation is required to understand the role of TERRA in cancer.

## 5. Targeting Telomeric Components in Cancer

Given the important and essential role of telomeres in cancer, various telomere targeting strategies have been designed and are currently under investigation for cancer therapeutics.

### 5.1. TRF1 Inhibitors

Telomere uncapping results in rapid cell death or senescence even in the absence of telomere shortening, suggesting that it can be exploited as a therapeutic strategy in cancer [180,181,182]. Altered expression of various shelterin components is observed in various cancers and implemented in various pro-tumorigenic properties (as described in Section 2). *TRF1* abrogation has been reported to cause acute telomere uncapping and along with *p53* deficiency, loss of *TRF1* promoted squamous cell carcinoma in mice. This suggested the role of TRF1 in tumor suppression. However, García-Beccaria and colleagues investigated the possibility of acute telomere uncapping phenotype associated with *TRF1* deletion could have a therapeutic effect in the *k-Ras^G12 V^* lung cancer mouse model [71]. They found that genetic deletion of *TRF1* resulted in impaired lung carcinogenesis and increased survival of mice even in the absence of *p53* tumor suppressor. Following the in vivo observations in mice, the group screened for various TRF1 chemical inhibitors and found two compounds, namely ETP-47228, ETP-47037 could disrupt TRF1 binding at the telomeres and demonstrated inhibition of *k-Ras^G12 V^* -induced *p53*-deficient lung tumor growth. Increased γH2AX foci and induction of TIFs were accompanied by decreased TRF1 foci in lung cancer cells upon treatment with these molecules. TRF1 inhibition caused telomere uncapping and induced telomere specific DNA damage. The induction of DNA damage and cell cycle arrest led to an impaired proliferation in the lung cancer cells, without having deleterious effects on the survival or viability of the mice [71]. TRF1 is overexpressed in patient-derived primary glioma stem cells (GSCs), tumors, and several glioblastoma mouse models. Poor prognosis in glioblastoma is attributed to high proliferation, cell heterogeneity, and GSCs. TRF1 chemical inhibitors have been reported to affect stemness independent of telomere length, killing tumor-initiating populations as well. Oral administration of TRF1 inhibitors resulted in decreased tumor growth in patient-derived xenograft models generated from primary GSCs and did not affect the cognitive function or neuromuscular dysfunction [70]. Thus, these inhibitors have promising therapeutic potential and demand further exploration. Clinical relevance of *TRF1* expression levels in predicting the outcome of different types of cancer should also be investigated to demonstrate TRF1 as a clinically relevant cancer target.

### 5.2. Telomerase Inhibitors

**Figure 2:**
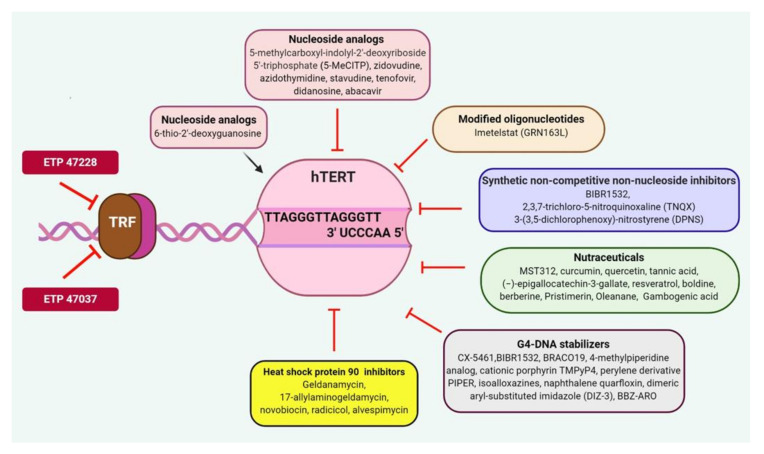
Targeting telomeric components and telomerase as a potential therapeutic approach against human malignancies. TRF1 inhibitors such as ETP-47228, ETP-47037 block the TRF1 binding at the site of telomeres and prevent the formation of a shelterin complex to eradicate cancer cells. Several classes of telomerase inhibitors including nucleoside analogs, oligonucleotides, nonnucleoside, nutraceuticals, Isothiazolone derivatives, G4-DNA stabilizer.

Telomerase represents a highly specific cancer target because normal cells either do not express telomerase or have minimal activity except in germ cells, stem cells, and immune cells. Inhibition of canonical as well as noncanonical activities of telomerase has been exploited against cancer. Telomerase inhibitors can be divided into several classes including nucleoside analogs, chemically modified oligonucleotides, synthetic mixed type noncompetitive nonnucleoside inhibitors, natural compounds, and derivatives, isothiazolone derivatives, G4 DNA stabilizers, and HSP90 inhibitors (Table 2 and Figure 2). The biochemical assay used to measure telomerase activity in vitro is PCR-based telomeric amplification protocol (TRAP) assay (Table 2). In vivo telomerase activity is observed by measuring telomere length posttreatment with these inhibitors using assays like Southern blot hybridization or by in situ hybridization using telomeric fluorescent probes and microscopy or flow cytometry.

#### 5.2.1. Nucleoside Analogs

Telomerase is a reverse transcriptase enzyme that uses deoxynucleoside triphosphates (dNTP) as a substrate to elongate telomeric DNA. Therefore, nucleoside analogs represent the most primitive inhibitors to be used to inhibit telomerase activity by being incorporated into telomeric DNA and blocking processivity of telomerase along with telomere dysfunction. Examples include zidovudine (Azidothymidine or AZT), stavudine, tenofovir, didanosine, and abacavir [183,184]. However, the major disadvantage is high in vivo toxicity and lack of efficacy in preclinical cancer models.

In addition, the major challenge for direct telomerase activity inhibitors is the lag period that is required to post continuous inhibitor treatment to achieve telomere shortening and replicative senescence of cancer cells. Overcoming these disadvantages, nucleoside analog 6-thio-2’-deoxyguanosine (6-thio-dG) has been reported as a promising telomerase dependent telomere-targeting therapeutic agent. The analog 6-thio-dG does not inhibit telomerase enzyme however telomerase preferentially utilizes it as a substrate thus incorporating it into telomeres and this accumulation of 6-thio-dG in telomeres causes telomere dysfunction only in telomerase positive cell lines resulting in their rapid cell death [185]. It was shown to be highly effective against primary NSCLCs and resistant NSCLCs [185,186]. The analog 6-thio-dG has been shown to be effective in inhibiting drug-resistant pediatric brain cancers [187]. The analog 6-thio-dG has also been reported to have promising antitumor activity in BRAF as well as checkpoint inhibitor-resistant melanomas [186].

Recently, an indole nucleotide analog, 5-methylcarboxyl-indolyl-2’-deoxyriboside 5′-triphosphate (5-MeCITP) has also been reported to function as an inhibitor of telomerase activity [188]. In vitro studies have reported that 5-MeCITP could function at similar potency as AZT to inhibit telomerase and was less cytotoxic than AZT. In addition, its nucleoside analog led to telomere shortening in telomerase positive cancer cell lines (Table 2 and Figure 2).

#### 5.2.2. Chemically Modified Oligonucleotides

Imetelstat (originally named as GRN163L) is a 13-mer oligonucleotide sequence composed of thio- phosphoramidate backbone and covalently bound 5′ palmitoyl (C16) lipid group. Imetelstat binds TERC with very high affinity and abrogates the interaction between telomerase and telomeric DNA. Mechanistically Imetelstat acts as a competitive telomerase template antagonist rather than eliciting its effect through antisense inhibition. This prevents telomere lengthening, resulting in gradual telomere shortening as cells undergo replication [189]. Thus, cell proliferation is inhibited only after a certain lag phase which is required to reach critically short telomeres. The thio-phosphoramidate backbone imparts resistance to the effect of cellular nucleases and provides stability in plasma and tissues and improves its binding affinity with its target. The lipid group boosts cell permeability to improve potency and enhance its pharmacokinetic and pharmacodynamic properties. In preclinical studies, imetelstat has been extensively studied for its activity and efficacy against several cancer cell lines and in mouse xenograft models. Imetelstat demonstrated potent inhibitory action against telomerase in a wide spectrum of cancers like lung [190], liver, esophagus [191], prostate, pancreas, breast [192], GBM [193] and hematological malignancies including multiple myeloma [194] and lymphoma [195]. Several studies showed the synergistic or additive effect of imetelstat when it is used in combination with existing cancer drugs or radiation. In HER2+ breast cancer imetelstat alone, and in combination with trastuzumab, decreases the cancer stem cell population and self-renewal of cells [196]. Based on in vitro and in vivo efficacy in a series of animal studies, imetelstat has entered multiple phase I/II clinical trials for several cancers. Table 3 summarizes the clinical trials of imetelstat on cancer patients.

#### 5.2.3. Chemically Synthesized Mixed Type Noncompetitive Nonnucleoside Inhibitors

Mixed type noncompetitive nonnucleoside class of inhibitors has mostly been identified using chemical library screening with the readout as inhibition in TRAP activity. These include BIBR1532; 2,3,7-trichloro-5-nitroquinoxaline (TNQX); 3-(3,5-dichlorophenoxy)-nitrostyrene (DPNS) and they inhibit telomerase-dependent telomere elongation by directly binding to telomerase [197,198,199]. The binding site for most of these inhibitors has been predicted to be away from the *TERC* or DNA template on TERT. None of these inhibitors have reached the clinical trial stage because of various disadvantages like the lag period before inhibiting cell proliferation, cytotoxicity at high doses, and limited bioavailability (Figure 2).

#### 5.2.4. Natural Compounds and Derivatives

Various natural compounds have reported to inhibit telomerase activity, induce telomere shortening, and affect cancer cell proliferation. These include polyphenols (e.g., curcumin, quercetin, tannic acid, (−)-epigallocatechin-3-gallate (EGCG) and resveratrol), alkaloids (e.g., boldine, berberine), triterpenoids (e.g., pristimerin, oleanane), xanthones (e.g., gambogic acid and gambogenic acid) [200]. These compounds have also been reported to possess antioxidant activity however their exact mode of action on telomerase is not known. Synthetic compounds have also been synthesized based on the natural compound structure which shows telomerase inhibition. One among them is EGCG derivative MST-312 which was shown to inhibit telomerase activity in various cancer cell lines while having minimal effect on normal cells [201]. Short-term treatment with MST-312 resulted in growth arrest in an ATM-dependent manner and increased apoptosis. Long-term (>1.5 months) exposure to MST-312 resulted in a shortening of telomeres in a promyelocytic leukemia cell line via suppression of the NF-κB pathway [202]. MST-312 treatment was reported to decrease telomerase activity, increase telomere dysfunction, and inhibited the growth of breast cancer cells [203].

#### 5.2.5. G4-DNA Stabilizers

From drug discovery and designing perspective, telomeric G4 is an attractive molecular target for anticancer therapeutics. G4-stabilizing compounds have been observed to inhibit telomerase activity, disrupt telomere capping, and telomere maintenance resulting in apoptosis [204,205]. They can also inhibit cell proliferation in a telomerase-independent manner in cancer cells with the ALT pathway [206,207,208]. Sun et al. initially reported a G4 interacting compound 2,6-diamido-anthraquinone analog that could inhibit telomerase activity [209]. Following this study, several ligands with telomeric G4-stabilizing and telomerase inhibiting properties have been synthesized and investigated including fluorenones, pentacyclic RHPS4, natural product telomestatin, substituted acridines like BRACO19 and 4-methylpiperidine analog, cationic porphyrin TMPyP4, perylene derivative PIPER, isoalloxazines, naphthalene BIBR1532 and quarfloxin [210]. In the past few years, many such novel ligands have been synthesized that show promising telomeric G4 stabilization and telomerase inhibition and subsequent retardation of cancer cell growth with varying levels of efficacy. A dimeric aryl-substituted imidazole (DIZ-3) inhibited cell proliferation in an ALT-positive cancer cell line U2OS, showing selectivity to multimeric G4 [211]. A synthetic β-carboline-benzimidazole derivative was found to be efficient in G4 DNA stabilization over double-stranded DNA, inhibited telomerase activity, and induced apoptosis in Hela cervical cancer cell line [212]. A dinuclear phenanthroline complex, [(dmb)_2_Ru(obip)Ru(dmb)_2_]^4+^ showed high affinity and specificity for various conformations of G4 DNA rather than double stranded DNA. In vitro coculture experiments demonstrated that [(dmb)_2_Ru(obip)Ru(dmb)_2_]^4+^ compound could specifically inhibit telomerase activity and cancer cell proliferation with no effect on normal fibroblast cells [213].

CX-5461, a potent rRNA synthesis inhibitor, selectively inhibits Pol I-driven transcription, DNA replication, and protein translation and could also function as an efficient G4 stabilizer in telomeric overhang repeats. It was found to repress *TERT* transcription thus reducing telomerase activity [214]. CX-5461 has been reported to inhibit tumor growth and is currently in advanced phase I clinical trial for patients with *BRCA1/2* deficient tumors (NCT02719977) [215]. A series of novel schizocommunin derivatives have also been observed to selectively stabilize and bind to the telomeric G4 in vitro as well as in cells. One such derivative named compound 16 was able to activate DDR at telomeric regions, induced telomere shortening and telomere uncapping, resulting in cell cycle arrest and apoptosis. Compound 16 was also found to inhibit tumor growth in a mouse xenograft model of cervical squamous cancer [216]. A disubstituted bisbenzimidazole naphthalenediimide (NDI) ligand BBZ-ARO, was reported to possess high telomeric G4 affinity, which could inhibit telomerase enzyme activity and caused G2/M arrest subsequently inducing apoptosis in cells with a good therapeutic index [217]. Divalent cationic naphthalene diimide ligands have been shown to selectively bind with telomeric G4 ligand and some of them could specifically kill cancer cells while having very less effect on normal cells [218].

A novel series of l0-(3,5-dimethoxy)benzyl-9(10H)-acridone derivatives have also been observed to stabilize telomeric G4 and act as antiproliferative agents [219]. Several platinum-based ligands have also been studied as telomeric G4 stabilizers that inhibit telomerase in cancer cells in vitro and in vivo [220,221,222].

TERRA has also been reported to form G-quadruplex dimer which could be developed as a structural target for anticancer agents directed against telomeres [223].

Although, structure-based drug design and in vitro and in vivo experiments have helped in the study of a number of telomeric G4 stabilizers and G4 themselves, it is crucial to determine different conformations of telomeric G-quadruplexes and their structure-specific locations and functions for ligands of appropriate specificity to be rationally selected for further investigation. The major disadvantage of G4 DNA stabilizers is their inherent affinity for nontelomeric G4 DNA which may be responsible for nonmalignant cytotoxic damage as has been observed in some studies [224].

#### 5.2.6. Heat Shock Protein 90 (HSP90) Inhibitors

HSP90 plays an important role in telomerase complex assembly and thus the available HSP90 inhibitors were tested for their effect on telomerase complex with the assumption that it may disrupt the complex [225]. HSP90 Inhibitors like geldanamycin, 17-allylaminogeldamycin, novobiocin, radicicol, and alvespimycin inhibit cancer cell proliferation however the effect may be nonspecific since HSP90 is known to function in various other signaling pathways [226].

### 5.3. Human TERT Targeting Immunotherapy

#### 5.3.1. Immunotherapy Using TERT-Derived Peptide Vaccines

**Figure 3:**
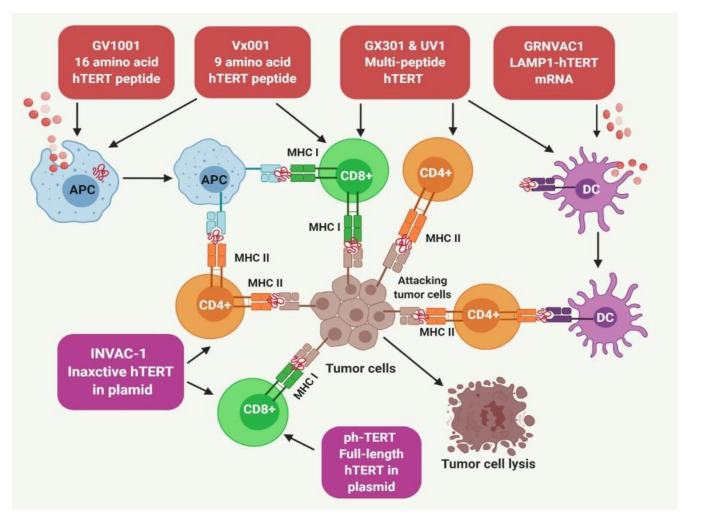
Telomerase-based immunotherapy and the role of the immune system in eliminating cancer cells. Several telomerase-based immunotherapies including peptides and plasmids have been developed to eradicate the tumor cells expressing human TERT peptides on their surface. These antigenic peptides are recognized by CD8+ and CD4+ T cells via the major histocompatibility complex class I and class II, respectively. This results in the amplification of telomerase-mediated cytotoxic T lymphocytes response in cancer patients. GV1001 has MHC class II-restricted hTERT peptide which is taken up by antigen-presenting cells (APCs) to represent it as an MHC class I peptide resulting in both CD4+ and CD8+ immune activation. GX30, UV1, and INVAC-1 produce both CD4+ and CD8+ immune response. GRNVAC1 and Vx001 trigger CD4+ and CD8+ T cells to eliminate hTERT-expressing cancer cells, respectively. hTERT: human telomerase reverse transcriptase; CD8: cluster of differentiation 8; CD4: cluster of differentiation 4; MHC: major histocompatibility complex.

Mizukoshi and Kaneko have reviewed several TERT-derived immunogenic peptides as targets for cancer immunotherapy [227,228]. Most vaccines that use these TERT-derived peptides were found to have major histocompatibility complex (MHC) I and MHC II epitopes specific to the tumor [229,230]. GV1001 and GRNVAC1 are two well-studied vaccines against human malignancies (Figure 3).

GV1001 is an MHC class II-restricted 16-mer peptide vaccine from the active site of TERT (611–626, EARPALLTSRLRFIPK) that needs granulocyte–macrophage colony-stimulating factor (GM-CSF) or toll-like receptor 7 (TLR-7) for CD4^+^ and CD8^+^ T-cell and cytotoxic T lymphocyte (CTL) activation [231,232]. GV1001 also acts on cells directly. Kim and colleagues showed that GV1001 gets localized in the cytoplasm after penetration through cell membranes and lowers the level of intracellular and surface HSPs (HSP90, HSP70) and HIF-1a and VEGF in tumor cells under hypoxic conditions [233,234]. There is experimental evidence of GV1001 inducing apoptosis in prostate cancer and renal carcinoma [235,236,237]. GV1001 was the first TERT peptide vaccine to be evaluated in clinical trials against advanced pancreatic cancer, lung carcinoma, melanoma, and liver carcinoma [227,232,238,239,240,241,242,243]. Another report evaluated the mode of action and found that GV1001-specific Th cells recognize only those antigen-presenting cells (APCs) that are internalized in the tumor and lymph nodes [232]. Recently, an observational study of 50 patients with solid cancer patients who were injected with GV1001 showed improved quality of life.

GX301 is an excellent example of a multi-peptide vaccine comprises of four TERT-specific peptides (TERT_540–548,611–626,672–686,766–780_) that can bind to both MHC class I and II. It also contains two adjuvants Montanide ISA-51 and Imiquimod [244]. Phase I trial of patients with stage IV prostate and kidney cancer demonstrated that all the patients manifested promising immune responses to at least one of the peptides and the overall response was more for the multi-peptide vaccines than single-peptide vaccines [245]. A phase II trial in a castration-resistant prostate cancer patient is currently ongoing [NCT02293707] (Figure 3).

UV1 is a multi-peptide vaccine consisting of three TERT specific peptides [TERT_691–705_ (RTFVLRVRAQDPPPE); hTERT_660–689_ (ALFSVLNYERARRPGLLGASVLGLDDIHRA; hTERT_652–665_ (AERLTSRVKALFSVL). In phase I and IIa trials, UV1 was administered along with GM-CSF for a period of six months in patients with prostate cancer representing metastasis. Seventeen of twenty-one patients (85.7%) registered an immune activation and 64% of the patients showed reduced levels of prostate-specific antigen (PSA). Postvaccination, magnetic resonance imaging analysis showed that tumor mass disappeared in 45% of patients with prostate cancer [246]. Currently, clinical trials for UV1 vaccination are ongoing in patients with NSCLC and melanoma [NCT01789099 NCT03538314, NCT02275416] (Figure 3).

Vx-001 is a bipeptide vaccine comprising of 9-mer cryptic TERT peptide along with optimized variant TERT peptide. The variant TERT peptide has tyrosine residue at the beginning to increase its affinity towards MHC class I [247,248]. The antitumor effect of Vx-001 was evaluated in lung cancer, breast cancer, bile duct tumor and melanoma in phase I/II clinical trials. Vx-001 evoked a strong immune response (TERT specific) and improved clinical outcomes in these clinical trials while causing acceptable toxicity side effects such as skin rashes [249,250,251,252,253,254,255] (Figure 3).

#### 5.3.2. TERT Targeting Dendritic Cells (DCs) for Immunotherapy

DCs are the most efficient APCs acting as a link between innate and adaptive immune systems. GRNVAC1 is a DC-based tumor vaccine and generated through the transfection of mature DCs with *TERT* mRNA and lysosomal associated membrane protein 1 (LAMP1) [256]. LAMP1 guides TERT into lysosomes to generate small peptides and antigenic epitopes. These peptides presented by DCs represent different portions of the TERT peptide to generate polyclonal immune responses [231,257]. The metastatic prostate cancer patients injected with GRNVAC1 developed robust CD4+ CTL response in comparison to patients who were administered DCs with nonchimeric hTERT. Patients treated with GRNVAC1 did not experience autoimmunity and the vaccine was well-tolerated even after repeated administration. Additionally, GRNVAC1 produced antigen-specific CD8+ and CD4+ T cells [256,258] (Figure 3). The administration of the vaccine for a long period has been found to be quite effective against AML [259].

GRNVAC2 also makes use of the DCs and is generated with a similar approach as GRNVAC1. GRNVAC2 employed human embryonic stem cells to produce DCs instead of monocytes for better delivery system [247,260]. GRNVAC2 has many more advantages over peptide vaccines because this vaccine solves the issue of human leukocyte antigen (HLA) mapping and may be effective in tumors with unknown T-cell epitopes. Several reports evaluated the different modes of administration for DC-based TERT vaccines. For instance, reports evaluated the efficacy of a vaccine where DCs were transfected with survivin mRNA or *TERT* tumor antigen mRNA along with silencing indoleamine 2,3-dioxygenase (IDO) expression, in patients with metastatic melanoma who had been previously treated with antiCTLA-4 blocking antibodies (ipilimumab). This vaccine-induced T-cell response against survivin as well as TERT. It also evoked T-cell-mediated immune responses against the melanoma-associated antigen recognized by T cells (MART-1) and NY-ESO-1 (New York Esophageal Squamous Cell Carcinoma-1) as detected in the peripheral blood. These vaccinated patients showed reduced metastases to different organs including lung, skin, liver with increased overall survival [261]. In another study by Frolkis and colleagues, adenovirus expressing *TERT* was generated and used for the transduction of DCs to induce TERT specific CTLs. In comparison to the plasmid-based system, the virus-based approach significantly increased the expression of *TERT* and then CTL responses [262].

Recently, novel emerging approaches where DCs were used for the generation of therapeutic grade dendritic-like cells known as tumor antigen-presenting (TAP) cells were described. In the studies evaluating TAP cell-based strategy, the vaccine was shown to improve the survival of patients with melanoma as well as increased the doubling time of PSA to elicit T-cell responses in prostate cancer patients. Moreover, approximately 60% of the patients showing delayed-type hypersensitivity (DTH) reactions against the lysates, indicating that the treatment promoted antitumor memory. In addition, this study reported that the TAP cell-based vaccine significantly expanded the number of T helper 1 (Th1) and T helper 17 (Th17) cells [263].

Mehrotra and colleagues generated a pulsed DC vaccine with three different HLA A2-restricted TERT peptides (TERT572Y), CEA (Cap1-6D), and survivin. This was used for the treatment of pancreatic cancer in a phase I trial. The treatment elicited specific T-cell responses with stable disease in 50% of the patients and medial overall survival of 7.7 months. The vaccine was well-tolerated, with the most common side effects being transient fatigue and flu-like symptoms [264].

In an adenovirus-based approach, a recombinant TERT adenovirus was constructed after conjugation of a recombinant antigen and mannan receptor of DC, which induced antigen-specific CTL response and antitumor effect in mice [265]. Similarly, a combination vaccine consisting of mannan-modified adenovirus that expresses both TERT and vascular endothelial growth factor receptor-2 (VEGFR-2) was created. It induced a potent antitumor immunity and inhibited intratumoral angiogenesis by activating CTL response against TERT and VEGFR-2 [266].

In phase I clinical trial, DCs transfected with *p53*, *survivin*, and *TERT* encoding mRNA in combination with mCy (metronomic regimen of cyclophosphamide) has been used in patients with progressive metastatic melanoma to evaluate the feasibility and safety profiling. The treatment was well-tolerated with manageable side effects and was shown to improve clinical outcomes [267].

#### 5.3.3. DNA Vaccines

With the emergence of recombinant DNA technology, TERT peptide can be improved to produce more efficient epitopes on the surface of APCs. The recombinant plasmids can be directly delivered to APC’s via electroporation and gene gun.

phTERT is an example of a DNA vaccine. phTERT contains full-length DNA against TERT. phTERT was first injected into murine and nonhuman primates through electroporation and triggers long-lasting and appreciable CD8+ T-cell response specific to TERT, which includes IFN-γ, TNF-α, and CD107a expression. Immunized monkeys showed strong IFN-γ and perforin release, indicating that phTERT exhibited potential cytotoxicity. A previous study with an HPV16-associated tumor model examined the prophylactic preventive as well as the therapeutic potential of the phTERT vaccine wherein reduced tumor growth and increased overall survival was observed [268,269,270] (Figure 3).

INVAC-1 is one of the DNA plasmid-based vaccines. INVAC-1 contains an inactive form of TERT. The electroporation-based administration of INVAC-1 has shown improved antitumor response in clinical trials. Studies in a mouse model showed that INVAC-1 induced TERT-specific T-cell responses, including CD4+ T cells and CD8+ T cells. INVAC-1 treatment has been reported to suppress the growth of tumors along with improving survival rate in approximately 50% of the HLA-A2 spontaneous mouse sarcoma model [271]. Recently, Teixeira and colleagues conducted a phase I study to investigate the safety, tolerability, clinical response, and immunogenicity of INVAC-1 in twenty-six patients with relapsed or refractory solid tumors with the administration via intradermal route followed by electroporation or by Tropis. This study demonstrated that INVAC-1 vaccination was safe, highly immunogenic when administered intradermally [271,272,273] (Figure 3).

#### 5.3.4. Cell-Based Approaches

Human umbilical vein endothelial cells (HUVECs) have been immortalized using TERT through lentiviral transduction approach. Modified HUVECs were irradiated to inhibit cellular growth and then subcutaneously injected into lung and colorectal cancer murine models where they maintained high telomerase activity and expressed CD31, integrin a5, and VEGFR-II. The vaccination has been shown to elicit both humoral and cellular immunity and developed antitumor immunity in murine models [274].

#### 5.3.5. Gene-Modified T-Cell Therapy

This involves genetic manipulation or engineering of T cells to generate T cell receptors (TCRs) that specifically recognize cancer antigens and their epitopes for successful cancer therapy [275,276]. There are two well-established techniques for generating genetically modified T cells; the first approach involves the utilization of tumor/cancer antigen-specific TCRs emerging from tumor-specific T cells and the second approach involves the generation of chimeric antigen receptors (CARs) whose extracellular region has a single-chain antigen recognition receptor consisting of the variable regions of a monoclonal antibody that specifically recognize the tumor-specific antigen while the intracellular region is composed of a costimulatory molecule that binds to the intracellular portion of the TCR [276,277,278,279]. TCR-engineered T (TCR-T) cells can be generated via modification of T cells genome to recognize the complexity of antigen peptides and MHC molecules expressed on the surface of cancer cells. Hence, TCR-T cell therapy could be efficient to eliminate the cancer cells expressing targeted antigenic epitopes and/or MHC molecules. It has been observed that several tumors express antigenic epitopes derived from TERT. Therefore, TCR-T immunotherapy that specifically targets these antigenic epitopes may be beneficial for treating human malignancies expressing TERT. To date, several reports have discovered TCRs that recognize hTERT and suggested that these TCRs can be utilized for immunotherapy [280,281,282].

#### 5.3.6. TERT-Targeted Cancer Immunotherapy: Challenges and Future Perspectives

Several appreciable advancements have been made to develop immunotherapies that specifically target TERT by employing hTERT DNA, peptides as well as DCs against human tumors. However, the effects of these immunotherapies were modest, but scientists are continuously working on these therapies to make them better. One of the reasons is that TERT is a self-antigen and to induce appreciable autoimmunity is quite difficult and challenging. Additionally, the antigen affinity of TCRs of induced T cells is low and this results in weak antitumor response. Generally, the induction of anticancer effects of these vaccines requires some time to manifest that could favor the activation of other adaptive responses like alternative lengthening of telomeres mechanism that necessitates the administration of a huge number of T cells having TCRs enable of exerting favorable anticancer activity [283]. No severe adverse effects have been noticed for TERT immunotherapy. Activation/induction or administration of tumor-specific T cells is essential for those patients where T cells are suppressed. Only activated T cells can infiltrate into tumors. Even then telomerase-targeted immunotherapy may be a reliable strategy and may be used in combination with immune checkpoint inhibitors or molecular targeted therapies for better efficacy in human tumors [284,285,286,287,288].

## 6. Conclusions

Advancements in genome sequencing and analysis technology have revealed the importance of telomeres and telomeric proteins in the initiation and progression of cancer. The deregulated expression of shelterin proteins has been observed in a variety of human malignancies and was associated with tumor progression, metastasis, maintenance of cancer stem cells, and drug resistance. Further, the discovery of germline mutations in shelterin proteins and their association with cancers has paved the way to understand their potential role in the process of malignancy. Moreover, the discovery of the TPMs was a seminal finding that was a conceptual advancement towards the role of transcriptional regulation instead of altering protein function as cancer-driving event. However, TPMs along with other genetic alterations in *TERT* contribute to telomerase activation only in a subset of cancers, and thus deciphering the mechanism of telomerase activation in remaining cancers remains unidentified.

Interestingly, telomerase activity is specifically high in cancer cells to ensure their immortality and several approaches have been investigated to exploit its therapeutic potential. However, specific inhibition of the telomerase activity results in a lag period due to telomere shortening before cells stop proliferating. This lag period reduces therapeutic efficacy and increases side effects. Thus, telomerase inhibitors targeting telomere elongation ability of telomerase may be more effective in cancers that have shorter telomeres. Nucleoside analog 6-thio-dG was found to overcome this problem of the lag period before inhibiting cell proliferation, due to its unique mode of action, and treatment with 6-thio-dG led to the rapid elimination of cancer by inducing programmed cell death. The G4 stabilizers have also emerged as inhibitors of telomerase and cause rapid cell death due to the induction of prominent telomere dysfunction. An additional advantage of G4-stabilizing compounds has been that they can be also effective against telomerase negative tumors since they target telomeric structure. However, G4 conformation occurs in various nontelomeric genomic regions apart from being present at telomeres. Thus, future efforts are required to develop G4 stabilizers specifically targeting telomeres and display cancer specificity. Another approach has been to target shelterin component TRF1 which has also shown promise in xenograft mouse models and functions independent of telomerase. There should be studies to understand which telomere or telomerase directed therapies would be useful to which subset of cancers by understanding their effect in association with the presence of other genetic alterations. Further, pharmacodynamic parameters alongside treatment with telomere or telomerase directed compounds in patients should also be investigated to determine the group of patients which will be responsive to the therapy and understand which parameters determine their efficacy.

Antitelomerase immunotherapies have emerged as an attractive approach. These therapies include hTERT peptide, DNA, DCs as well as genetically engineered T cells against human tumors. While studies on antitumor immune mechanisms have advanced tremendously, it can be envisaged that no single immunotherapy would be sufficient to eliminate cancer. Thus, developing novel approaches that employ a combination of various strategies may continue to achieve improved survival of cancer patients in the future.

## Figures and Tables

**Table 1:** Differential expression of shelterin subunits in human malignancies/diseases.

Shelterin Subunit	Disease Reported with Upregulation	Ref.	Disease Reported with Downregulation	Ref.	Disease Reported with the Genetic Mutation	Ref.
TRF1	Hepatocellular carcinoma Lung cancer Gastric carcinoma Acute lymphoblastic leukemia T-cell leukemia Renal cell carcinoma Prostate cancer Glioblastoma	[55] [56] [57] [58] [59] [60] [61] [70]	Gastric cancer Acute myeloid leukemia B-chronic lymphocytic leukemia Breast cancer Mesenchymal sarcomas	[24] [72] [73] [74] [75]		
TRF2	Hepatocellular carcinoma Lung cancer Gastric carcinoma T-cell leukemia Renal cell carcinoma Prostate cancer Skin carcinoma	[55] [56] [57,62] [59] [60] [61] [91]	Acute myeloid leukemia Breast cancer Gastric cancer	[72] [74] [76]	Chronic lymphocytic leukemia	[78]
RAP1	Gastric carcinoma Multiple myeloma Renal cell carcinoma	[62] [63] [77]	B-chronic lymphocytic leukemia	[73]	Melanoma	[91]
TIN2	Hepatocellular carcinoma T-cell leukemia Multiple myeloma	[55] [59] [63]	Acute myeloid leukemia Gastric cancer	[72,76]		
POT1	Multiple myeloma Gastric cancer Laryngeal cancer	[63] [64] [65]	B-chronic lymphocytic leukemia Mesenchymal sarcomas	[73,75]	Angiosarcoma Glioma Mantle cell lymphoma Chronic lymphocytic leukemia Melanoma	[82,85] [85,86] [87] [79,89,90] [80,81,85,91]
ACD	Multiple myeloma Laryngeal cancer B-chronic lymphocytic leukemia	[63] [65] [73]			Chronic lymphocytic leukemia Melanoma	[79] [91]

TRF1: telomere repeat factor 1, TRF2: telomere repeat factor 2, RAP1: repressor/activator protein 1, TIN2: TRF interacting nuclear protein 2, POT1: protection of telomere 1, ACD: adrenocortical dysplasia protein homolog.

**Table 2:** Several types or classes of telomerase inhibitors and their mechanism.

Different Types or Classes of Telomerase Inhibitors	Name of Drugs or Agents	Mechanism of Their Action	Identification Methods	Outcomes and Safety Profile	References
Nucleoside analogs	AZT, stavudine, tenofovir, didanosine and abacavir 6-thio-dG, 5-MeCITP	Incorporate into telomeric DNA to prevents the addition of dNTP and telomerase activity resulting into impairment of telomere except for 6-thio-dG	TRAP method as well as direct telomerase assay were used for validation	Lower efficacy in preclinical cancer models as well as associated with toxicity and nonspecific Less cytotoxic than AZT	[183,184,185,186,187,188]
Modified oligonucleotide	Imetelstat (GRN163L) 13-mer oligonucleotide sequence with thio- phosphoramidate and palmitoyl lipid group	Robustly binds to the human telomerase RNA (hTR) template to hamper its recruitment to telomeric DNA leading telomerase inhibition and shortening of telomeric ends	TRAP method was used for validation	Suppress cellular and tumor growth Limited toxicity in phase I/II clinical trials	[189,190,191,192,193,194,195,196]
Synthetic mixed noncompetitive nonnucleoside inhibitor	BIBR1532 TNQX (2,3,7-trichloro-5-nitroquinoxaline), DPNS (3,5-dichlorophenoxy-nitrostyrene)	Suppress telomerase dependent telomere lengthening	TRAP method was used for validation.	Suppress cellular growth and induce cell death High doses were associated with cytotoxicity	[197,198,199]
Nutraceuticals	MST-312, EGCG, curcumin, quercetin, tannic acid, rhodacyanine, genistein, resveratrol, gambogic acid boldine, gambogenic acid oleanane, berberine, pristimerin	Suppress telomerase activity and telomere shortening	Nutraceuticals and their derivatives were validated via TRAP assay	Reduced tumor growth in a preclinical model Lower stability and bioavailability	[200,201,202,203]
Isothiazolone derivatives	TMPI	Isothiazolone moiety may bind with the sulfhydryl of cysteines in the active site of the TERT to attenuate telomerase enzymatic activity	High-throughput using the TRAP method discovered isothiazolone derivatives including TMPI	No data for effects on cancer cell proliferation	[197,198,199]
G4-DNA stabilizers	CX-5461, BIBR1532, telomestatin, RHPS4, BRACO-19 and TMPyP4, fluorenones, 4-methylpiperidine analog, perylene derivative PIPER, isoalloxazines, quarfloxin naphthalene, TERRA, BBZ-ARO	G-quadruplex has displayed to suppress telomerase activity and telomeric elongation	TRAP method was used for the validation of G-quadruplex stabilizers in blocking the telomere elongation.	Limited stability, pharmacokinetics Bind nonspecifically to g-quadruplex in the promoter and other regions in the genome associated with off-target effects	[204,205,206,207,208,209,210,211,212,213,214,215,216,217,218,219,220,221] [222,223,224]
HSP90 inhibitors	Geldanamycin, 17-allylaminogeldamycin, novobiocin, radicicol, and alvespimycin	Hamper the assemble of telomerase	Small molecule inhibitors against HSP90 were verified using TRAP assay	Inhibit cellular growth and induce apoptosis of cancer cells	[225,226]

TRAP: telomeric repeat amplification protocol; AZT: azidothymidine; 6-thio-dG: 6-thio-2′-deoxyguanosine; 5-MeCITP: 5-methylcarboxyl-indolyl-2 0 -deoxyriboside 5 0 -triphosphate.; EGCG: Epigallocatechin gallate; TMPI: 2-[3-(trifluoromethyl)phenyl]isothiazolin-3-one; HSP: heat shock protein.

**Table 3:** Details of imetelstat (GRN163L: telomerase inhibitor) in clinical trials against several human malignancies.

Clinical Trial Identifier	Phases	Human Malignancies/Conditions	Objective	Design	Results
NCT00594126	I	Refractory or relapsed multiple myeloma	Evaluation of maximum tolerated dose (MTD) and safety profile	3 + 3 cohort; dose escalation study	Dose limiting toxicity (DLT): anemia, thrombocytopenia, neutropenia, a PTT prolongation, fatigue, nausea, anorexia dizziness
NCT00732056I	I	Recurrent or metastatic breast cancer	Evaluation of MTD and safety profile Efficacy in combination with paclitaxel and bevacizumab	3 + 3 cohort; dose escalation study	DLT including thrombocytopenia and neutropenia
NCT00310895	I	Refractory or relapsed solid tumors	Evaluation of MTD and safety profile	3 + 3 cohort; dose escalation study	DLT including thrombocytopenia and myelosuppression
NCT 00718601	I	Multiple myeloma	Evaluation of MTD and safety profile. Efficacy in combination with bortezomib and dexamethasone	3 + 3 cohort; dose escalation study	Results are unavailable
NCT00124189	I	Refractory chronic lymphoproliferative disease	Evaluation of MTD, safety, tolerability, DLT	Sequential dose cohort, open label, escalation trial evaluating one infusion duration of 2 h; weekly intravenous infusion	Results are unavailable
NCT00510445	I	Non-small cell lung cancer with metastasis	Evaluation of safety, DLT, MTD in combination with a standard paclitaxel/ carboplatin regimen	Dose cohorts with a minimum of three patients	Patients with imetelstat plus short autologous tumor lysate (TL) displayed longer median progression free survival (PFS) and overall survival (OS). On the other hand, imetelstat plus long TL had no improvement in median PFS or OS Adverse drug reactions (ADRs) includes neutropenia, and thrombocytopenia
NCT01265927	I	HER2+ breast cancer	Evaluation of DLT in combination with trastuzumab	Open label, nonrandomized study	Results are unavailable
NCT01242930	II	Multiple myeloma	Improved outcome in patients previously treated with imetelstat	Imetelstat 2 h intravenous Infusion on day 1 and day 8 of a 28-day cycle	Results are unavailable
NCT02426086	II	Patients with myelofibrosis and previously treated with JAK inhibitors	Evaluation of safety and efficacy	Randomized, single-blind, multicenter	Recruiting patients
NCT01731951	II	Primary or secondary Myelofibrosis	Efficacy	Open label, parallel, active, not recruiting	Complete or partial remission in 21% patients. Bone marrow fibrosis was reversed in a few patients.
NCT01243073	II	Essential thrombocythemia	Evaluation of safety and efficacy	Open label, single group	Eighteen patients and all with positive hematologic response. Positive molecular response in most patients with JAK2 V617 F mutation. ADRs includes neutropenia, anemia
NCT02598661	III	Myelodysplastic syndrome	Safety and efficacy	Randomized, double-blind	Recruiting patients
